# Senescence and cell death in chronic liver injury: roles and mechanisms underlying hepatocarcinogenesis

**DOI:** 10.18632/oncotarget.23622

**Published:** 2017-12-22

**Authors:** Mengchao Xiao, Wenjian Chen, Chao Wang, Yingfu Wu, Shiwei Zhu, Chuyang Zeng, Yongchao Cai, Changcheng Liu, Zhiying He

**Affiliations:** ^1^ Department of Cell Biology, Center for Stem Cell and Medicine, Second Military Medical University, Shanghai, China; ^2^ Institute for Regenerative Medicine, Shanghai East Hospital, School of Life Sciences and Technology, Tongji University, Shanghai, China

**Keywords:** chronic liver injury, senescence, apoptosis, necroptosis, necrosis

## Abstract

Chronic liver injury (CLI) is a complex pathological process typically characterized by progressive destruction and regeneration of liver parenchymal cells due to diverse risk factors such as alcohol abuse, drug toxicity, viral infection, and genetic metabolic disorders. When the damage to hepatocytes is mild, the liver can regenerate itself and restore to the normal state; when the damage is irreparable, hepatocytes would undergo senescence or various forms of death including apoptosis, necrosis and necroptosis. These pathological changes not only promote the progression of the existing hepatopathies via various underlying mechanisms but are closely associated with hepatocarcinogenesis. In this review, we discuss the pathological changes that hepatocytes undergo during CLI, and their roles and mechanisms in the progression of hepatopathies and hepatocarcinogenesis. We also give a brief introduction about some animal models currently used for the research of CLI and progress in the research of CLI.

## INTRODUCTION

With population growth and aging, the incidence of cancer has become increasingly high. According to the statistics of the GLOBOCAN, the number of new cases of cancer and cancer-related deaths in the world is about 14.1 million and 8.2 million respectively in 2012, including 782,500 new cases of liver cancer and 745,500 liver cancer-related deaths [1]. Hepatocellular carcinomas (HCC) has become an important concern, especially in China, where both the number of new cases of HCC and the number of HCC-related deaths account for about 50% of the global figures. HCC in China ranks the 5th and 9th most common cancer in men and women respectively [1]. The understanding about mechanisms underlying hepatocarcinogenesis will provide a theoretical basis for the clinical treatment of this devastating disease. With the increased incidence of chronic alcoholic liver disease, fatty liver, viral hepatitis and other chronic liver diseases, the role of CLI in the pathogenesis of HCC has aroused even greater concern.

Pathological change of hepatocytes varies with the degree of CLI. When the damage to hepatocytes is mild, the liver can repair and remodel itself and then restore to the normal state; but when the damage to hepatocytes is out of control and most hepatocytes undergo necrosis, acute liver failure may occur. When the liver exhausts all its intrinsic proliferation potentials (known as replicative exhaustion or replicative senescence), or is confronted with some acute exogenous and endogenous stress, hepatocytes will undergo senescence and present a senescence-associated secretory phenotype (SASP), finally resulting in significant changes in the microenvironment and tissue homeostasis [2]. Apoptosis is a common presentation when hepatocytes are subjected to alcoholic stimulation, cholestasis and viral infection [3–5]. Apoptosis is usually associated with the severity of the liver disease and participates in the formation of liver fibrosis [6]. When apoptosis of hepatocytes is inhibited, such as in the case of invasion by the viral gene that expresses anti-apoptotic proteins, necroptosis as a backup pathway will be activated, as represented by swelling of cellular organelles and cytoplasm with subsequent rupture of the plasma membrane and cell lysis [7].

The above research findings suggest that there is some relationship between CLI-induced cellular senescence and apoptosis and necroptosis. For instance, necroptosis or apoptosis induced by activation of death receptors depends on two kinases: receptor-interacting protein 1 (RIP1) and receptor-interacting protein 3 (RIP3) [8–10]. Activation of Caspase-8 expression can make cells more susceptible to apoptosis rather non-necroptosis by depolymerizing the complex of RIP1 and RIP3, while Caspase-8 inhibition will promote the assembly of the RIP1/RIP3 complex, forming necrosomes, which are known as key substances for necroptosis signaling transduction that promotes cells to progress to necroptosis [11, 12]. Under common circumstances, apoptosis is a response to great stress, while senescence occurs when cells experience a relatively small damage. Other than the degree of stress, the balance between the pre-senescence and pre-apoptosis signaling pathways can also determine the fate of cells. For example, upper stream signaling controls the acetylation balance of lys residues on p53. Low-level p53 with lys residue acetylation at K161/K162 site would promote cell cycle arrest and senescence. While down-regulated p53 level with lys residue acetylation at K117 site would induce transcription of the apoptosis-promoting gene, resulting in cell apoptosis [13]. Pre-senescent cells will positively present the anti-senescence phenotype, while senescent cells have the capability of inhibiting apoptosis [14]. Cellular senescence, apoptosis and necroptosis are closely associated with tumorigenesis and progression.

## CELLULAR SENESCENCE AND TUMORIGENESIS

### Characteristics of cellular senescence

Cellular senescence is a program in response to various sources of cell stress like oxidative stress or oncogene activity [15, 16], and this process can restrain damaged cells from proliferating and ensure a stable state of proliferative arrest, subsequently altering the microenvironment and tissue homeostasis [2]. Senescence-associated β-galactosidase (SA-β-gal) is a senescence-related content, which increases with age [17], and is overexpressed within senescent cells [18]. Senescent cells also secrete various pro-inflammatory cytokines, chemokines, growth factors and proteases. This process is known as the SASP, the most outstanding feature of cellular senescence [19], because it explains the role of cellular senescence in biological senescence and senescence-related pathology [20]. In the nuclei of partial senescent cells, there exist senescence-associated heterochromatic foci (SAHF) and senescence-associated DNA-damage foci (SADF). The former activate proliferation-associated genes [21], and the latter contain proteins that play key roles in DNA-damage stress and cellular senescence [22]. Meanwhile, cellular senescence also plays a critical role of anti-tumorigenesis in different environments and tissues [23–25]. Anti-cancer therapy can induce premature senility of primarily cultured cells or cancer cells, which is known as therapy-induced senescence (TIS) [26, 27].

### Hepatocyte senescence and hepatocarcinogenesis

The number of senescent hepatocytes in the liver increases with age as we reported previously [28]. Hepatocyte senescence is characterized by expression of SA-β-gal activity, blockage of cell proliferation, accumulation of foci of DNA damage and increased levels of cell cycle inhibitors p16^INK4A^, p21 and p53 [28]. Senescence of hepatocytes can also be induced by metabolic stress, oncogene over-expression or deletion of tumor suppressing genes, either *in vivo* or *in vitro* [29–31]. SASP can promote immune surveillance on senescent cells and further clear up senescent cells in the tissues. When the immune system fails to execute surveillance in normal tissues, senescent cells generally accumulate in the liver. For instance, patients with co-infection of hepatitis C virus (HCV) and human immunodeficiency virus (impaired CD4^+^ T-cell function) often showed accumulation of p16-positive hepatocytes, a consequence of deficiency in immune cell function. Accumulation of massive senescent hepatocytes was also observed in the liver of patients who received immunosuppressive therapy after liver transplantation for HCV-related liver cirrhosis [29, 32, 33]. There is a close relation between hepatocyte senescence and hepatocarcinogenesis. Over-expression of Nras^G12V^ [neuroblastoma RAS viral (v-Ras) oncogene homolog] could promote senescence of hepatocytes in a HCC mouse model, thus inhibiting HCC initiation. The mechanism lies in the ability of senescent cells to activate T helper type 1 (Th-1) cells, which in turn specifically identify oncogene products expressed by senescent cells, thus mediating monocytes/macrophages to execute the clearance of pre-malignant senescent hepatocytes [30]. In the same animal model, Eggert et al. [34] found that Nras^G12V^ over-expression could induce senescent hepatocytes to secrete chemokine C-C motif ligand 2 (CCL2) to recruit immature CCR2^+^ myeloid cells. These immature CCR2^+^ myeloid cells differentiated to macrophages, which helped Th-1 cells clear up pre-malignant senescent cells. In addition, recruitment and activation of pro-inflammatory immune mediators such as M1 macrophages, Th-1 lymphocytes and NK cells can drive the clearance of senescent tumor cells, thus further inhibiting tumorigenesis. Also, Th-1 lymphocytes secrete IFN-γ, which is generally believed to play a key role in antagonizing tumor growth [35, 36].

Macrophages can also drive the senescence surveillance. Tumor-associated macrophages are recognized as the important component in the tumor microenvironment, among which M1 polarized macrophages could promote tumor evacuation [37]. It was also found that senescent hepatic stellate cells (HSC) expressing p53 protein could release regulatory factors under the condition of CLI, which induced macrophages to differentiate to tumor-inhibitory M1 polarized macrophages to evacuate senescent cells, thus forming an anti-tumor microenvironment. Therefore, CLI-induced hepatocarcinogenesis involved inflammatory response and powerful recruitment of immunocyte populations by senescent hepatocytes [38].

Although the main function of cellular senescence is to inhibit tumorigenesis, it also has much to do with tumorigenesis. Several studies demonstrated that SASP could activate inflammatory responses, promote cell proliferation and lead to initiation of HCC at least in some microenvironments [39]. Researchers found that in patients with HCC, senescent peri-tumor tissues induced accumulation of CCR2^+^ myeloid cells and then inhibited NK cell function in a manner of SASP secretion. Consequently, inhibition of NK cells facilitated the progression of HCC [34]. It was found that senescent fibroblasts could stimulate the proliferation of human or mouse malignant epithelial cells when they were co-injected into immunodeficient mice, while non-senescent fibroblasts did not show this effect [40]. This proliferation-promoting effect was believed to be due to soluble factors generated by senescent cells [41], among which matrix metalloproteinase 3 (MMP3) as the component of SASP is especially important [40]. MMP3 promotes epithelial-mesenchymal transition (EMT) and mammary carcinogenesis [42, 43]. With the presence of DNA damage, SASP induces the EMT of pre-malignant cells and enhances their invasiveness. Among all these components, IL-6 and IL-8 plays a major role in such induction [19]. IL-6 activates transcription factor STAT3, phosphorylates Jun-(N)-terminal Kinase (JNK) and ERK, indirectly alters AKT and mammalian target of rapamycin (mTOR)-S6K signaling, and ultimately promotes hepatocarcinogenesis in an obesity-induced chronic inflammatory microenvironment [44]. Another study also found that diet- or gene-induced obesity could alter the gut microbiota, thus causing elevation of deoxycholic acid (DCA), which is known to be a bacterial metabolite contributing to DNA damage. DCA can cause DNA damage and induce senescence of hepatic stellate cells, while senescent hepatic stellate cells can secrete SASP factors. SASP contains various inflammatory and tumor-promoting factors, such as IL-6, Gro-α and CXCL9, and enhances the promoting effect on hepatocarcinogenesis of HSC. When mice were exposed to chemical carcinogenic factors, existing SASP also promoted the development and progression of HCC [45]. Other SASP components secreted by non-parenchymal cells like HGF, KGF and HB-EGF stimulated hepatocyte DNA synthesis potently and showed a correlation with tumorigenesis [46]. Studies on hepatocyte senescence and hepatocarcinogenesis are listed in Table 1.

**Table 1:** Studies on hepatocyte senescence and hepatocarcinogenesis

Models	Cytokines or key proteins	Outcome	Mechanisms	References
Nras^G12V^-transfected mice	CCL2	tumor suppression or promotion	Senescence-induced CCL2-CCR2 signaling and the ensuing myeloid cell accumulation have distinct functions in preventing HCC initiation, but also in promoting progression of established HCC	[34]
Nras^G12V^-transfected mice	IL-1α	tumor suppression	Antigen-specific CD4^+^ T cells secret IL-1α to exert the function of senescence surveillance	[29]
Mdr2^-/-^Rage^-/-^mice	RAGE	tumor promotion	RAGE regulates oval cell activation and promotes tumor development	[87]
Obesity-associated HCC mice	senescence secretome	tumor promotion	DCA–SASP axis promotes HCC	[45]
p53^LoxP/LoxP^ conditional KO mice	p53	tumor suppression	p53 suppress tumorigenesis by promoting an antitumor microenvironment	[38]
p53^-/-^INK4a/ARF ^-/-^ compound mutant mice	AKT p53	tumor suppression	AKT-driven tumors undergo senescence in vivo following p53 reactivation	[32]

### Hepatocyte senescence and liver injury

Hepatocytes senescence also involves in development and progression of other chronic liver diseases besides HCC. Aravinthan et al. found that under non-alcohol-related fatty liver disease (NAFLD), hepatocytes underwent senescence and exhibited such characteristics as telomere shortening, DNA damage, permanent cell cycle arrest and elevated p21 expression [47]. Telomere shortening in the liver was also observed after HCV infection, leading to replicative senescence. It was found that the fibrosis stage of patients with chronic hepatitis C was significantly correlated with senescent cell accumulation [48]. Such senescence occurs highly selectively in hepatocytes rather in hepatic stellate cells or lymphocytes, and this process is associated with progression of liver cirrhosis [49].

## APOPTOSIS AND TUMORIGENESIS

### Apoptosis in the liver

Cell apoptosis is a highly controllable biochemical process mediated by caspases, during which cells and a greater proportion of their components are dissembled into fragments [50, 51]. In NAFLD, hepatocyte apoptosis plays a critical role during the progression from mild steatosis to NAFLD [3]. In the liver of patients with nonalcoholic steatohepatitis (NASH), free fatty acid upregulates the expression of tumor necrosis factor (TNF)-related apoptosis-inducing ligand (TRAIL) death receptor 2 (TRAIL-R2) expression in hepatocytes through activating JNK signaling, and then turns hepatocytes from TRAIL cytotoxicity resistant to sensitivity. It is one of the ways through which TRAIL plays its significant role in steatosis and liver injury [52]. In the liver with chronic hepatitis B virus (HBV) infection, the TRAIL expression in NK cells was increased together with the expression of TRAIL-R2 on HBV-specific CD8^+^ T cells. This upregulation promotes NK cells to delete HBV-specific CD8^+^ T cells by inducing T cell apoptosis, and finally limits the virus-specific T cell response [53]. Besides, confirmed apoptosis effector genes, like the p53 up-regulated modulator of apoptosis (PUMA, a BCL-2 family member) and Bim, were found to be activated in the NASH liver [54]. As apoptotic cells can be evacuated quickly, apoptosis is regarded as a non-inflammatory or low-grade inflammatory process. Activation of cells undergoing apoptosis depends on two signaling pathways. The intrinsic pathway induces apoptosis mainly through alteration of the mitochondrial outer membrane permeability mediated by members of the BCL-2 family, release of cytochrome C and activation of caspases [55]. Hepatocyte-specific deletion of BCL-x_L_, an anti-apoptosis BCL-2 family protein, leads to continuous hepatocyte apoptosis, oxidative stress and higher levels of inflammatory cytokines. TGF-β, one of these cytokines produced by non-apoptotic hepatocytes and macrophages that engulf apoptotic hepatocytes, directly conducts an intralobular fibrogenic response. Another cytokine TNF-α, in accompany with oxidative stress, is correlated with apoptosis-induced hepatocarcinogenesis [56, 57]. What’s more, BCL-x_L_ controls the pathway switch between senescence and apoptosis. Silencing or inhibition of BCL-W and BCL-x_L_ triggers the apoptosis of senescent cells which were resistant to apoptosis. This means that the BCL protein family members play an essential in apoptosis resistance [58, 59]. Endoplasmic reticulum (ER) stress, p53 activation and other apoptosis triggering factors can also activate this pathway. Intracellular accumulation of free fatty acid, as well as viral infection, induces ER stress [60, 61]. For instance, in a NASH mice model under high-fat diet, sustained ER stress in hepatocytes activated SREBP1, contributing to lipogenesis and steatosis. Hepatocytic steatosis and ER stress increase reactive oxygen species (ROS) production and cause oxidative stress, thus inducing genomic instability and consequently leading to hepatocarcinogenesis [62]. In addition, p53 is an important regulator of another intrinsic pathway. It can make responses to oncogene activation, DNA damage and senescence, and maintain itself in a stress state to regulate specific target genes such as Bax transcription, thus inducing apoptosis [63]. The external cell death pathway is usually provoked by members of the TNF family of death receptor ligands [64]. The death receptors are the main mediators in the apoptosis pathway and involved in the pathogenesis of many chronic liver diseases [65].

### Hepatocyte apoptosis and hepatocarcinogenesis

Hepatocarcinogenesis is closely associated with apoptosis. Studies have demonstrated that apoptosis shows opposite actions in transformed and non-transformed hepatocytes. The tumor-promoting effect of apoptosis in non-transformed hepatocytes has been clearly elucidated. Deletion of anti-apoptosis proteins such as Mcl-1 or BCL-x_L_ specifically in hepatocytes can not only accelerate the rate of hepatocyte apoptosis but induce the initiation and progression of spontaneous HCC during chronically increased apoptosis. *BCL-x*L or *Mcl-1*knockout (KO) mice exhibited increased myeloid-derived cell infiltration/activation, higher TNF-α release and oxidative stress in the liver, all of which promote cellular transformation or carcinogenesis [56]. In a hepatocyte-specific BCL-x_L_ knockout model, simultaneous knockout of Bak expression inhibited HCC initiation, thus excluding the effect of other BCL-x_L_ pathways on the initiation and progression of HCC, and providing direct evidence to support the relationship between hepatocyte apoptosis and hepatocarcinogenesis [65]. The result in another model showed that inhibition of NF-κB expression by conditional deletion of Nemo gene in hepatocytes could cause massive death of hepatocytes and induce the initiation of spontaneous HCC, which also confirms the relationship between hepatocyte apoptosis and hepatocarcinogenesis. Increased production of carcinogens due to deletion of hepatocyte-specific IκB kinase β (IKKβ) could induce cell apoptosis and compensatory proliferation of hepatocytes, thus promoting hepatocarcinogenesis [66]. On the contrary, knockout of the PUMA of the BCL-2 family would decrease the compensatory proliferation of hepatocytes [67]. Similarly, antibody-mediated Fas ligand neutralization could not only prevent hepatocyte apoptosis but inhibit the development of HCC in a HCC mouse model induced by HBsAg transgene [68].

Apoptosis promotes tumorigenesis in non-transformed hepatocytes but inhibits it in transformed hepatocytes. Mutation or deletion of certain molecules that regulate apoptosis signaling pathways could reverse this inhibitory effect and induce tumorigenesis in these cells. Tumor cells often undergo a process of selection; for instance, p53 mutant tumor cells evade apoptosis after escaping from the process of selection [69]. Receptor-interacting protein kinase 1 (RIPK1) participates in the regulation of multiple cell death and inflammation pathways. Research showed that RIPK1 deletion could induce TNF-mediated hepatocyte apoptosis without affecting the expression of NF-κB, and at the same time RIPK1 deletion in liver parenchymal cells promoted degradation of TNF receptor-associated factor 2 (TRAF2) resulting in liver damage, suggesting that deletion of RIPK1 and degradation of TRAF2 together promoted the development of HCC [70]. Shimizu et al. [71] found that linear ubiquitin chain assembly complex (LUBAC) could suppress carcinogenesis by inhibiting the initiation of apoptosis, and that LUBAC deletion in liver parenchymal cells could cause apoptosis of large numbers of hepatocytes because of increased sensitivity to apoptosis signaling, and subsequently induce the hepatocarcinogenesis in response to TNF receptor 1 (TNFR-1) medicated inflammatory response. Vucur et al. [72] discovered that apoptosis of liver parenchymal cells was a common phenomenon in patients with viral hepatitis and alcoholic or nonalcoholic steatohepatitis. Therefore, it may be highly significant to study the mechanism of using caspase molecules to inhibit transformation from chronic hepatitis to HCC in patients with chronic hepatitis C or NASH, like the caspase molecular inhibitors in a report [73, 74]. Studies on hepatocyte apoptosis and hepatocarcinogenesis are listed in Table 2.

**Table 2:** Studies on hepatocyte apoptosis and hepatocarcinogenesis

Model	Key genes	Outcome	Mechanisms	References
Ikkβ^Δhep^ mice	IKKβ	tumor promotion	IKKβ promotes hepatocarcinogenesis via cytokine-driven compensatory proliferation	[66]
BCL-x_L_ KO mice Mcl-x_L_ KO mice	TNF-α	tumor promotion	Deletion of Bak significantly inhibited hepatocyte apoptosis and suppressed HCC	[56]
HBV transgenic Mice	FasL	tumor suppression	Neutralization of the activity of Fas ligand prevented hepatocyte apoptosis, proliferation and liver inflammation, thus suppressing HCC	[68]
Mcl-1^Δhep^ mice	Mcl-1	tumor promotion	Hepatocyte-specific Mcl-1 deletion triggers proliferation and hepatocarcinogenesis	[56]
PUMA KO mice	PUMA	tumor promotion	JNK1/PUMA-dependent apoptosis promotes hepatocarcinogenesis via compensatory proliferation	[67]
RIPK1^LPC–KO^ Mice	RIPK1 TRAF2	tumor suppression	RIPK1 deficiency enhances TNF-induced TRAF2 degradation, leading to promote hepatocarcinogenesis	[70]
Hoip^flox^ mice	LUBAC	tumor suppression	LUBAC restrains TNFR1-independent apoptosis, suppressing hepatocarcinogenesis	[71]
TAK1^LPC-KO^ mice NEMO^LPC-KO^ mice	NEMO	tumor suppression	TAK1 suppresses a NEMO-dependent pathway, thus suppressing hepatocarcinogenesis	[82]

## NECROPTOSIS AND TUMORIGENESIS

### Characteristics of necroptosis

Necroptosis is a type of programmed necrosis as a defensive mechanism against endogenous pathogens and intracellular infection, sharing the same upstream pathway with apoptosis [75, 76]. It is currently believed that necroptosis is initiated as a candidate pathway when apoptosis is inhibited in such conditions as hepatocytes transfected by virus genes that express anti-apoptosis proteins [7]. The occurrence of necroptosis is mainly mediated by the TNF receptor superfamily, T cell receptor, interferon receptor, Toll-like receptor, cellular metabolism, genotoxic stress, and various anti-cancer chemicals [76]. Studies demonstrated that if expression of intracellular caspase-8 was inhibited after activation of the death receptor, the RIP1/RIP3 complex would assemble into necrosomes, and these necrosomes are the key transformers of necroptosis signaling [11, 12]. RIP3 was reported to be upregulated in human NASH, and RIP3-dependent necroptosis is an important pathway that regulates the fibrosis progression. This pathway can be suppressed by caspase-8 [77]. Mixed lineage kinase-domain like protein (MLKL) is the key mediator of necroptosis. Some researchers supposed that MLKL could increase the generation of mitochondrial ROS via the mitochondrial target [78]. In human autoimmune hepatitis, MLKL expression is upregulated and activated, and its upregulation is correlated with a translocation to the membranes. But both MLKL activation and translocation occur independently of RIPK3 activity [79]. Compared with other organs, a low level of RIP3 is found in the liver of healthy mammals [10]. However, the level of RIP3 was up-regulated in cells that became sensitive to necroptosis after Caspase-8 knockout [72]. Infection of mice with vaccinia virus would induce assembly of the RIP1/RIP3 complex in the liver, indicating that necroptosis participated in the anti-viral response *in vivo* [80].

### Necroptosis and hepatocarcinogenesis

In liver diseases, RIP3-dependent necroptosis is mainly involved in regulating the progression from NASH to NAFLD and NASH-induced liver fibrosis. For instance, in NASH a positive feedback loop is established between the elevated phosphorylation and activation of the kinase JNK and RIP3 expression, and the overexpressed RIP3 promotes inflammation, monocytes/macrophage recruitment and caspase-8-dependent necroptosis [77]. TGF-beta-activated kinase 1 (TAK1) activates NF-κB and JNK, and plays an essential role in maintaining hepatocellular homeostasis. Spontaneous hepatocyte death, compensatory proliferation, inflammation, fibrosis and hepatocarcinogenesis were observed in a mouse model with hepatocyte-specific Tak1 deficiency [81]. Some studies demonstrated that necroptosis could counteract apoptosis in hepatocytes lacking TAK1 [72, 82], wild-type hepatocytes and adipocytes [83]. Studies also demonstrated that dying cells would release damage-associated molecular patterns (DAMP), which is believed to induce sterile inflammation after tissue damage [84]. As DAMP can be released only when the integrity of the plasma membrane is damaged, DAMP release mainly occurs during apoptosis and necroptosis [85]. DAMPs like high-mobility group box 1 protein (HMGB1), toll-like receptor, methyl polypeptide, FPR1, ATP, P2X7 and DAMP receptors can induce recruitment of inflammatory cells in the liver, thus aggravating the damage [86]. A study [87] showed that receptor for advanced glycation endproducts (RAGE) as one of the HMGB1 receptors was closely associated with the proliferation of hepatic oval cells, indicating that RAGE may be a connection between DAMPs and hepatocarcinogenesis in CLI settings. It was found in a genetic model of CLI [72] that activation of RIP3 could restrain immune response and compensatory proliferation of liver parenchymal cells by inactivating Caspase-8 dependent JNK in liver parenchymal and non-parenchymal cells. It was also found in their study that RIP3 inhibited the intrahepatic tumor growth and prevented the Caspase-8 dependent specific chromosome from undergoing aberration, knowing that this aberration can mediate resistance to TNF-induced apoptosis, thus inducing hepatocarcinogenesis. Above all, the role of necroptosis in hepatocarcinogenesis remains elusive at present.

## NECROSIS

Necrosis is one of the prominent features in acute liver injury, as well as apoptosis [88]. Necrosis is regarded as an uncontrollable consequence occurring upon physiochemical stress, as represented by mitochondrial damage, ATP exhaustion and subsequent ATPase malfunction, leading to quick swelling of cells and organelles accompanied with formation of membrane “blebs”, and ultimately cell rupture [89]. As a result, the cell components overflow to the extracellular fluid, causing powerful inflammatory response. For this reason, necrosis is also regarded as an immunogenic form of cell death [85]. For instance, release of HMGB1 and heparin binding growth factor (HDGF) can induce inflammatory response mediated by the immune system [90, 91]. When the tissue is damaged, DAMP released from dying cells can induce sterile inflammation [84], while DAMP release mainly occurs after necrosis and necroptosis, which explains the inflammatory nature of cellular necrosis [85]. Several DAMPs and their receptors in the liver, such as HMGB1, formyl peptide or ATP, can all induce recruitment of inflammatory cells, finally aggravating liver injury [92, 93]. In addition, hepatocytes under stress release IL-33. It is currently believed that DAMP can promote hepatocarcinogenesis, and IL-33 can promote liver fibrosis [87, 94, 95]. Another DAMP component IL-1α activates IL-1R/MyD88 signaling in Kupffer cells, leading to IL-6 production and release, which promotes hepatocarcinogenesis [96]. Altogether, apoptosis, necroptosis and necrosis may co-exist in both acute and chronic liver injuries.

## RESEARCH UPDATES IN CLI MODELS

Construction of animal models is of great significance for the research of CLI in that it can heighten our awareness about the pathogenesis of CLI. The development of novel therapeutic methods, prognostic monitoring devices and therapeutic tools also depends on experimental animal models. However, no single animal model is currently available to display all features of human liver disease. As all animal models under current research and development can only mimic a certain feature of human liver disease, selection of an appropriate animal model is of primary importance.

Among various animal models, the use of mouse models is especially suitable because of their small size, relatively short life span and a short pregnant period, easy management and artificial reproduction. Furthermore, mice and human genes share significant similarities. CLI models mainly include the CCl_4_ model, chronic alcoholic liver damage model, genetic liver damage model, bile duct ligation-induced liver damage model, dimethylnitrosamine-induced liver damage model, and metabolic dysfunctional Fah^-/-^ mouse model. For instance, dimethylnitrosamine is a carcinogenic reagent, leading to DNA damage, oxidative stress and malignant transformation mainly in the liver, and the sex difference in the incidence of HCC is similar to that in humans [97, 98]. In a CCl_4_ model, CCl_4_ showed toxicity in the liver after it was metabolized to trichloromethyl radicals and trichloromethyl peroxy radical. The peroxy radicals attacked membrane lipids in a chain reaction manner, resulting in breakdown of the membrane [99]. CCl_4_ toxicity induces inflammation, oxidative stress, hepatocyte necrosis, regeneration, and consequently hepatocarcinogenesis. This process involves release of cytokines and interleukins by Kupffer cells after toxic chemical exposure [100, 101].

The Fah^-/-^ mouse model is a tyrosinemia type I model established by Grompe et al. with the gene of fumarylacetoacetate hydrolase (Fah) knockout (Fah^-/-^) [102]. Fah^-/-^ mice are hereditarily defective in degrading fumarylacetoacetate (FAA), leading to accumulation of toxic metabolites like FAA, maleylacetoacetate, and succinylacetone. Toxic metabolites induce progressive hepatocyte injuries like mitotic abnormalities and genomic instability, which lead to cell death and spontaneous hepatocarcinogenesis [103, 104]. Meanwhile, Fah^-/-^ mice can be rescued by oral administration of 2-(2-N-4-trifluoromethylbenzoyl)-1,3-cyclohexanedione (NTBC), a specific inhibitor of 4-hydroxyphenylpyruvatedioxygenase in tyrosine metabolic pathway that blocks the production FAA [105]. This feature makes Fah^-/-^ mice a highly controllability model of acute and chronic liver injury. Many studies have used the Fah^-/-^ mouse model to study the association between hepatocyte apoptosis and HCC. Besides, combined with the immunodeficiency model, researchers can actualize robust human hepatocyte xenografts on Fah^-/-^/Rag2^-/-^/Il2rg^-/-^ mice [106]. Some studies compared the occurrence rate of liver disease and hepatocarcinogenesis in immunocompromised or immunocompetent Fah^-/-^ mice, and found that although the death rate in immunocompromised Fah^-/-^ mice was high, the initiation of HCC was inhibited markedly, indicating that the immune system plays a unique role in liver regeneration and hepatocarcinogenesis [107]. For instance, a study reported that the CLI in Fah^-/-^ mouse model induced the resistance of cell death to promote hepatocarcinogenesis by activating the AKT pathway and inhibiting endogenous hepatocyte apoptosis [103]. Vogel et al. [108] found that chronic liver disease in Fah^-/-^ mice could induce cell death resistance, and stress-induced apoptotic dysfunction could promote the accumulation of damaged cells, thus increasing the risk of cancer. Later, Vogel et al. [109] also found that the phosphorylation state of BID (BH3 interacting-domain death agonist) determined the level of hepatocyte apoptosis, and that apoptotic resistance under chronic cholestasis may induce the risk of carcinogenesis in the long run. Orlik et al. [110] found that BID failed to regulate hepatocyte proliferation under the condition of CLI in the Fah^-/-^ mouse model, and did not participate in the DNA damage response in hepatocytes or HCC cells. On the contrary, BID promoted hepatocarcinogenesis by inhibiting the activity of p38. Researchers also found that the degree of liver damage and the intensity of p21 activation determined their impact on liver regeneration and the initiation and progression of HCC in the Fah^-/-^ mouse model. Sestrin2 is a small molecule identified from this model, and was found to be located in the crosslink of mitogenic mTOR, nuclear factor (erythroid-derived 2)-like 2 (Nrf2) and p53/p21 signaling network. Sestrin2 activation regulated hepatocyte proliferation and tumor development after liver damage in mice [111]. In addition, Willenbring et al. [112] found that the anti-proliferation function of p21 was necessary for inhibiting carcinogenesis in the CLI setting in the Fah^-/-^ mouse model, and this function could not be compensated by apoptosis. Our research team also used the Fah^-/-^ mouse model to investigate the association between hepatocyte senescence and hepatocarcinogenesis. We found that hepatocyte senescence appeared in Fah^-/-^ mice under acute liver injury, while hepatocyte senescence was inhibited in Fah^-/-^ mice under chronic liver injury with the consequence of a significant incidence of HCC. We postulate that inhibition of hepatocyte senescence promoted hepatocarcinogenesis in Fah^-/-^ mice (data unpublished). Obviously, selection of an appropriate rat or mouse model of liver damage according to the purpose of research is a prerequisite for the proper understanding of the post-CLI pathological change of hepatocytes, the underlying molecular mechanism and the role of CLI in hepatocarcinogenesis. It is also of great assistance for seeking effective strategies for the treatment of various chronic liver diseases.

## SUMMARY AND PROSPECTS

With the better understanding about the role and mechanism of CLI in hepatocarcinogenesis in recent years, senescence and cell death have been recognized to play a central role in hepatocarcinogenesis during the pathological process of CLI. Increased numbers of studies have also discovered new patterns of death such as necroptosis and some specific cell death-regulating pathways. These studies have helped us better understand the pathological process of CLI. Nevertheless, we are still unable to transfer them to clinical applications at present, which inspires us to beware the importance and necessity of effective cooperation between clinicians and scientists who are undertaking experimental research. Our future research should focus more on cellular senescence and death pathways, knowing that these pathways can provide important clues for the precision medical treatment of liver diseases. It is convinced that with constant in-depth research, new molecular markers for cellular senescence and death will be identified, thus enabling us to predict the pathological outcome and make therapeutic decisions more precisely.
